# Meloxicam inhibits STING phosphorylation and alleviates intracellular DNA-mediated autoimmune responses

**DOI:** 10.1186/s13578-023-01025-3

**Published:** 2023-04-29

**Authors:** Yu Yu, Miao Wang, Xiao-Wei Li, Jie Mao, Ying-Jie Zhu, Na Wang, Le-Hua Yin, Zeng-Lin Guo, Hong Cai, Tao Li, Ting-Ting Liang, Jiuwei Cui, Tao Zhou

**Affiliations:** 1 Cancer Research Institute of Jilin University, The First Hospital of Jilin University, Changchun, 130021 Jilin Province China; 2 Nanhu Laboratory, National Center of Biomedical Analysis, Beijing, 100850 China

**Keywords:** Meloxicam, Interferon, STING, TREX1, Autoimmunity

## Abstract

**Background:**

Cyclic GMP-AMP synthase (cGAS)-stimulator of interferon genes (STING) pathway is critical for cytosolic DNA-sensing and the subsequent immune responses. The inappropriate activation of this pathway leads to DNA-induced autoimmune response. Understanding the precise regulation of cGAS-STING pathway is important for developing therapeutics to treat several autoimmune diseases caused by self-DNA.

**Results:**

We report that Meloxicam (MXC) inhibits intracellular DNA-, but not RNA-induced immune responses. We find that MXC inhibits the phosphorylation of STING by examining in different cells with various DNA stimulations. We further find that MXC significantly dampens the expression levels of interferon-stimulated genes (ISGs) by using DNA 3’ repair exonuclease 1 (TREX1)-deficient cell, an experimental model for self-DNA-induced autoimmune disease. Importantly, we demonstrate that MXC could promote the survival in *Trex1*^*–/–*^ mouse model for Aicardi-Goutières syndrome (AGS).

**Conclusions:**

Our study identified a non-steroidal anti-inflammatory drug, MXC, that exhibits potential effect in treating the autoimmunity caused by self-DNA.

**Supplementary Information:**

The online version contains supplementary material available at 10.1186/s13578-023-01025-3.

## Background

Detection of microbial infections by sensing intracellular nucleic acids is an integral component of innate immunity [1]. cGAS is the key intracellular DNA sensor that detects foreign DNA or abnormal self-DNA and mediates the synthesis of the second messenger 2′3′-cyclic GMP-AMP (cGAMP) [2]. cGAMP then binds and activates STING on endoplasmic reticulum membrane to further recruit TANK-binding kinase 1 (TBK1), which facilitates the phosphorylation of STING and interferon regulatory factor 3 (IRF3) [3]. These molecular events lead to the production of type I interferon (IFN) and proinflammatory cytokines and thus play a critical role in inflammation [4].

Unbalanced and continuous production of type I IFN was observed in many autoimmune diseases [5, 6], and AGS is one of them [7]. The loss-of-function mutation of TREX1 have been frequently reported in AGS patients [8]. TREX1 is a DNase that mainly expressed in cytoplasm, and is responsible for the degradation of cytosolic DNA [9]. The deficiency of TREX1 leads to the accumulation of self-DNA in cytoplasm, which is an important danger signal for infection. Under such conditions, chronic inflammation persists and causes the detrimental effects in AGS [10]. Previous studies revealed that the natural chemical epigallocatechin gallate or aspirin could inhibit the activation of cGAS in cells from TREX1-deficienct AGS patients and experimental models and thereby alleviated the disease phenotypes of AGS [11, 12]. Thus, targeting the activation of cGAS-STING signaling is a potential treatment for self-DNA-induced autoimmune diseases.

Besides the usage in relieving pain and inflammation [13], non-steroidal anti-inflammatory drugs (NSAIDs) are also showed to be effective in the treatment of autoimmune inflammatory diseases [12, 14]. These findings inspire us to explore the potential role of other NSAIDs in targeting key molecules in the driving signaling pathways of autoimmune diseases, such as cGAS-STING pathway. In the current study, we show that a widely used NSAID, MXC, could suppress the intracellular DNA-mediated autoimmunity and inhibit the phosphorylation of STING. Our study presents MXC as a possible therapy for cGAS-STING-mediated autoimmune responses.

## Results

### MXC dampens intracellular DNA- but not RNA-sensing pathway

**Fig. 1:**
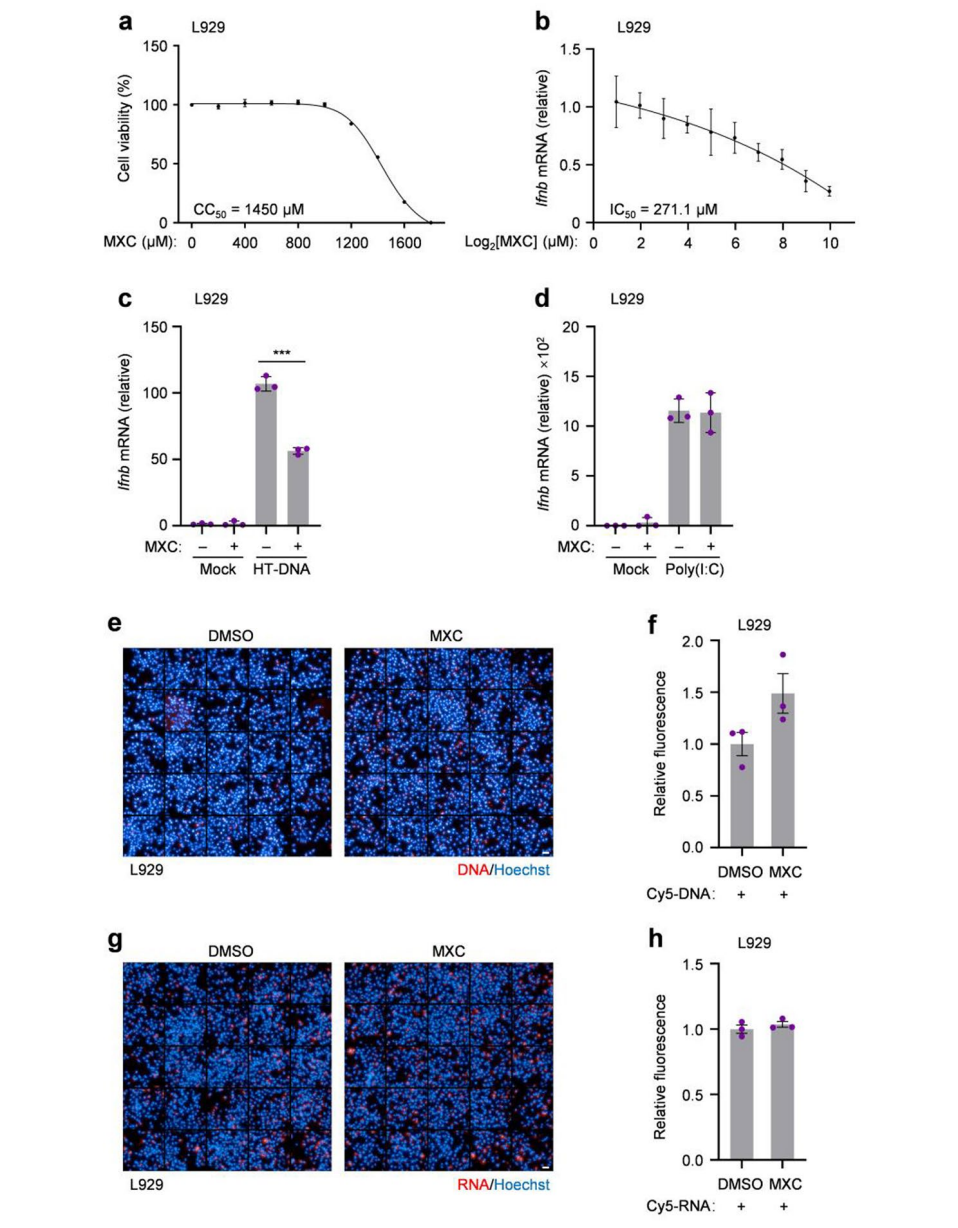
MXC dampens intracellular DNA- but not RNA-sensing pathway. **a** L929 cells were seeded in 96-well plates and cultured in the presence of MXC at indicated concentrations for 24 h and CC_50_ was calculated from the curve after nonlinear regression analysis. **b** L929 cells were pretreated with different concentrations of MXC for 6 h and then treated with 0.5 µg/ml HT-DNA for 3 h. The mRNA level of *Ifnb* was analyzed by qPCR and IC_50_ was calculated from the curve after nonlinear regression analysis. **c**, **d** qPCR analysis of *Ifnb* mRNA expression in L929 cells treated with HT-DNA (0.5 µg/ml) (c) or Poly(I:C) (1 µg/ml) (d) for 3 h following a 6 h of pretreatment with 500 µM MXC. **e** Representative fluorescent images of L929 cells transfected with Cy5-labeled DNA (1 µg/ml) for 3 h following a 6 h of pretreatment with 500 µM MXC. Scale bar, 50 μm. **f** Relative fluorescence of Cy5-labeled DNA per cell was quantified from 25 views, n = 3 biological replicates. **g** Representative fluorescent images of L929 cells transfected with Cy5-labeled RNA (1 µg/ml) for 3 h following a 6 h of pretreatment with 500 µM MXC. Scale bar, 50 μm. **h** Relative fluorescence of Cy5-labeled RNA per cell was quantified from 25 views, n = 3 biological replicates. Data are presented as the mean ± s.e.m. ***P < 0.001. Data represent at least three biological independent experiments

MXC is one of the oxicam-type NSAIDs, which was previously reported to be used to treat the experimental autoimmune animal models [15, 16]. We therefore sought to determine the role of MXC in the nucleic acids-triggered immune responses. To test the cytotoxicity of MXC, different concentrations of MXC were used to treat L929 cells, cell viability was measured 24 h after the treatment. Our results suggested that less than 1000 µM MXC is considered safe for L929 cells and the 50% cytotoxic concentration (CC_50_) is 1450 µM (Fig. 1a). We also applied L929 cells with different concentrations of MXC underlying herring testis DNA (HT-DNA) treatment, we found that the detected interferon-beta (*Ifnb*) mRNA level decreases as the MXC concentration increases and the 50% inhibitory concentration (IC_50_) is 271.1 mΜ (Fig. 1b). Then, we pretreated L929 cells with 500 µM MXC and treated with HT-DNA and RNA mimic polyinosinic:polycytidylic acid [Poly(I:C)] to trigger the intracellular nucleic acids-mediated immune responses measured by detecting the *Ifnb* mRNA level (Fig. 1c, d). The results showed that MXC is only involved in DNA- but not the RNA-sensing pathway. To rule out the possibility that MXC may affect the transfection, we then used the high-content confocal microscope to detect the fluorescence of transfected Cyanine 5 (Cy5)-labeled DNA or RNA. The results showed that MXC could not inhibit the transfection of nucleic acids (Fig. 1e-h). Hence, we focused on the DNA-sensing pathway to investigate the function of MXC in immune responses.

### MXC inhibits intracellular DNA-induced immune responses

**Fig. 2:**
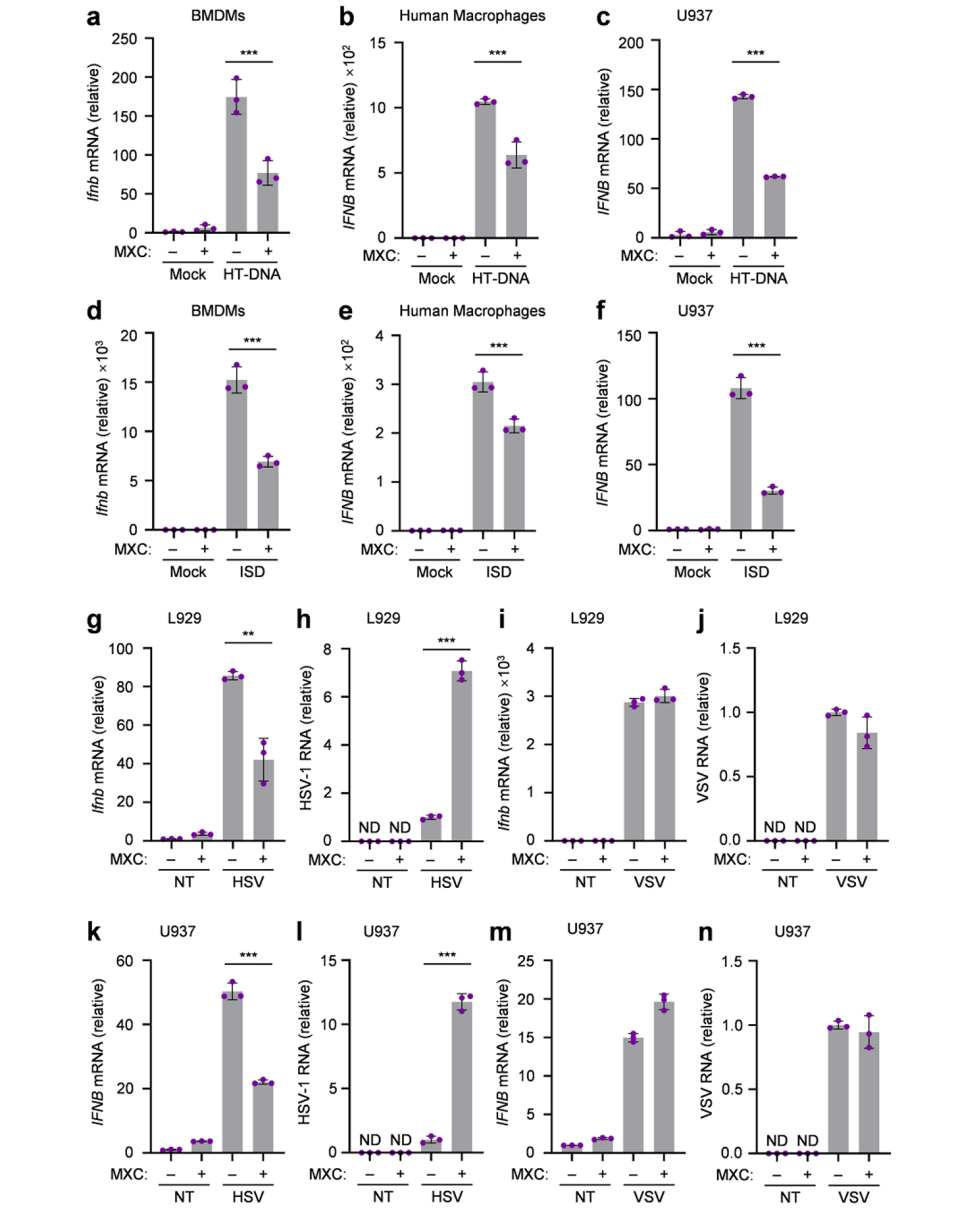
MXC inhibits intracellular DNA-induced immune responses. **a**, **b**, **c** qPCR analysis of interferon-beta mRNA expression in BMDMs (a), human macrophages (b) and U937 cells (c) treated with 0.5 µg/ml HT-DNA for 3 h following a 6 h of pretreatment with 500 µM MXC. **d**, **e**, **f** qPCR analysis of interferon-beta mRNA expression in BMDMs (d), human macrophages (e) and U937 cells (f) treated with 2 µg/ml ISD for 3 h following a 6 h of pretreatment with 500 µM MXC. **g**, **h** qPCR analysis of *Ifnb* mRNA expression (g) and HSV-1 RNA (h) in L929 cells that were untreated or infected with HSV-1 (MOI = 5) for 4 h. **i**, **j** qPCR analysis of *Ifnb* mRNA expression (i) and VSV RNA (j) in L929 cells that were untreated or infected with VSV (MOI = 1) for 4 h. **k**, **l** qPCR analysis of *IFNB* mRNA expression (k) and HSV-1 RNA (l) in U937 cells that were untreated or infected with HSV-1 (MOI = 5) for 4 h. **m**, **n** qPCR analysis of *IFNB* mRNA expression (m) and VSV RNA (n) in U937 cells that were untreated or infected with VSV (MOI = 1) for 4 h. NT, untreated. ND, not detected. Data are presented as the mean ± s.e.m. **P < 0.01, ***P < 0.001. Data represent at least three biological independent experiments

To confirm the above findings in DNA-sensing pathway, we used different cell types and stimulations to study the effect of MXC. We first isolated mouse bone marrow cells and differentiated them into bone marrow-derived macrophages (BMDMs), and the type I IFN production after HT-DNA transfection was suppressed by MXC (Fig. 2a). Then, human primary macrophages differentiated from peripheral blood mononuclear cells (PBMCs) and phorbol 12-myristate 13-acetate (PMA)-differentiated U937 cells were tested, and MXC was also showed to inhibit IFN expression in human cells (Fig. 2b, c). Consistent results were obtained when treated with interferon-stimulatory DNA (ISD) (Fig. 2d-f). Sensing nucleic acids is critical for host immune defense against viruses, such as herpes simplex virus 1 (HSV-1) [17] and vesicular stomatitis virus (VSV) [18]. We first infected cells with DNA virus HSV-1 or RNA virus VSV in L929 cells (Fig. 2g-n). The results showed that MXC could attenuate the type I IFN production induced by HSV-1, but not VSV (Fig. 2g, i). The abundance of viral RNA in L929 cells at 4 h after HSV-1 infection was much higher than that in untreated cells, which was not obvious under VSV infection (Fig. 2h, j). Moreover, we obtained the similar results after the infection of U937 cells with HSV-1 and VSV (Fig. 2k-n). Collectively, our data suggested that MXC is a useful drug for inhibiting the intracellular DNA-induced immune responses.

### MXC does not inhibit cGAS activation

**Fig. 3:**
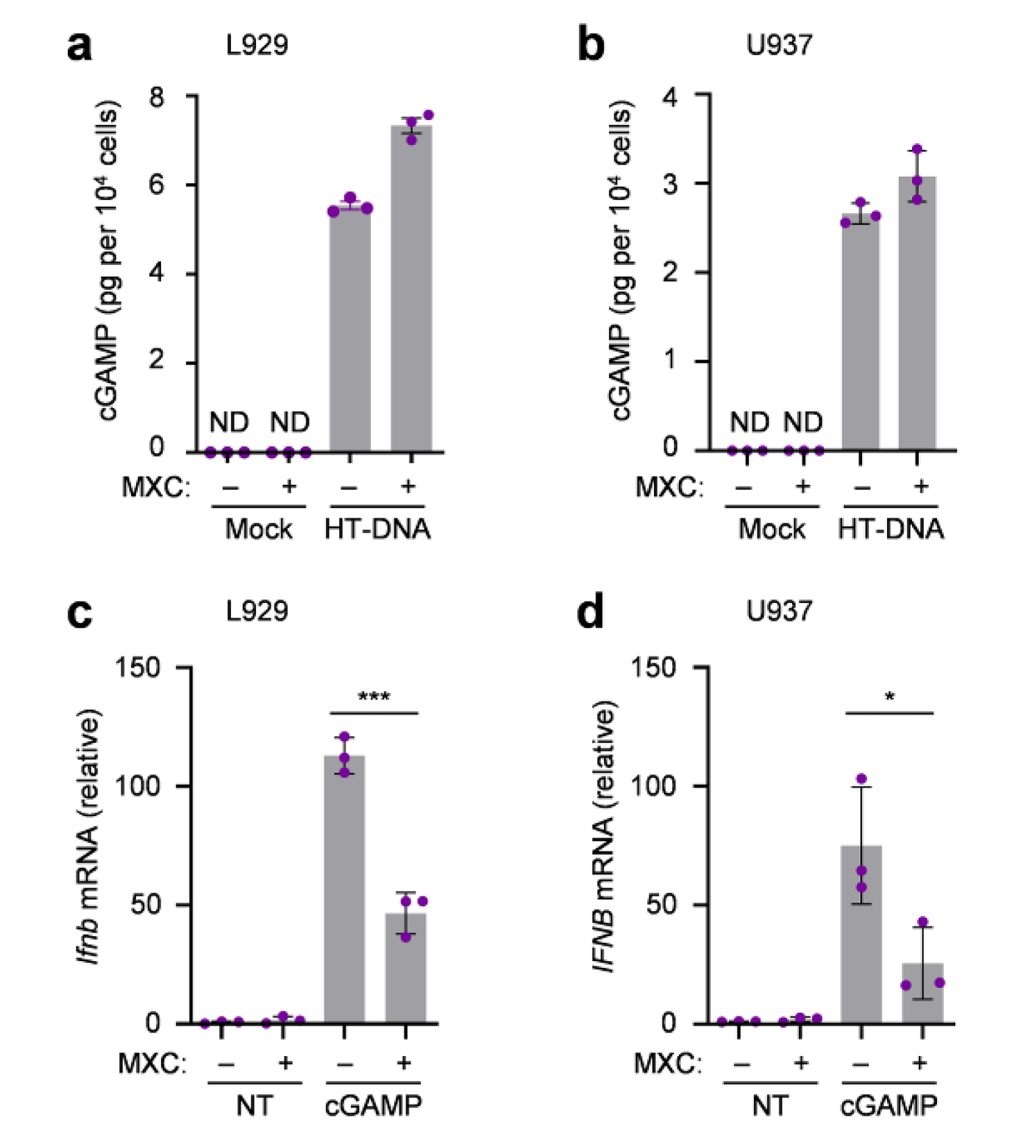
MXC does not inhibit cGAS activation. **a**, **b** Mass spectroscopy analysis of cGAMP production of L929 cells (a) and U937 (b) cells treated with 0.5 µg/ml HT-DNA for 3 h following a 6 h of pretreatment with 500 µM MXC. ND, not detected. **c**, **d** qPCR analysis of interferon-beta mRNA expression in L929 cells (c) and U937 cells (d) treated with 1 µg/ml cGAMP for 1 h following a 6 h of pretreatment with 500 µM MXC. NT, untreated. ND, not detected. Data are presented as the mean ± s.e.m. *P < 0.05, ***P < 0.001. Data represent at least three biological independent experiments

To further understand the mechanism of MXC in regulating DNA-mediated immune responses, we first examined the activation of DNA sensor cGAS by quantitating the synthesized cGAMP. Our data suggested that MXC could not suppress the cGAMP synthesis in the existence of intracellular DNA in both L929 cells and U937 cells (Fig. 3a, b). Next, we treated the cells with cGAMP to investigate the downstream pathway of cGAS. The results showed that MXC still inhibited the type I IFN production under cGAMP treatment in both indicated cell lines (Fig. 3c, d). Thus, our data indicated that cGAS is not responsible for the effect of MXC in intracellular DNA-induced immune responses

### MXC inhibits the phosphorylation of STING

**Fig. 4:**
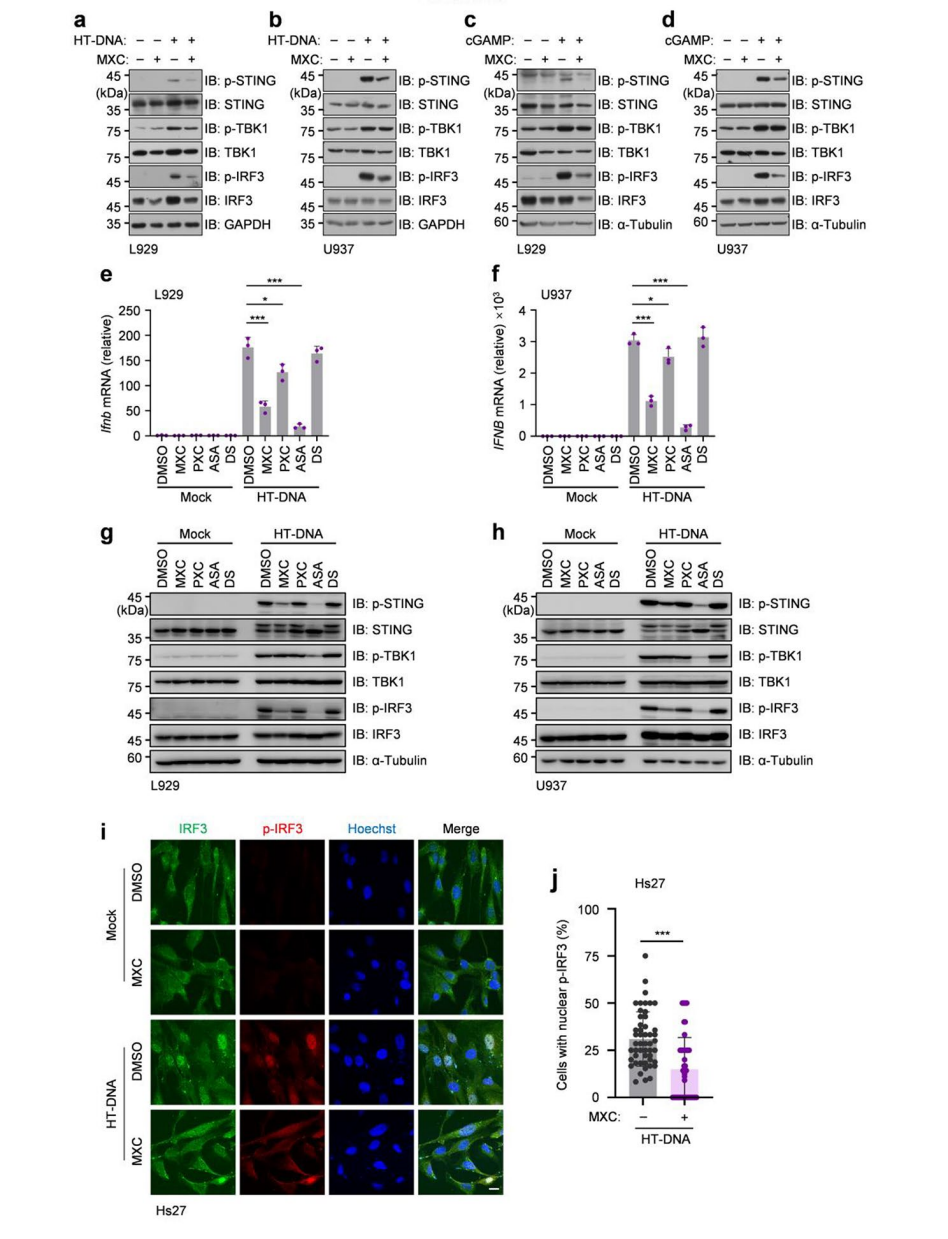
MXC inhibits the phosphorylation of STING. **a**, **b** L929 cells (a) and U937 cells (b) were treated with 0.5 µg/ml HT‑DNA for 3 h following a 6 h of pretreatment with 500 µM MXC and the cell lysates were immunoblotted with indicated antibodies. **c**, **d** L929 cells (a) and U937 cells (b) were treated with 1 µg/ml cGAMP for 1 h following a 6 h of pretreatment with 500 µM MXC and the cell lysates were immunoblotted with indicated antibodies. **e**, **f** L929 cells (e) and U937 cells (f) were pretreated with 500 µM MXC for 6 h, 250 µM PXC for 6 h, ASA for 24 h or DS for 24 h and then treated with 0.5 µg/ml HT‑DNA for 3 h. The mRNA level of interferon-beta was analyzed by qPCR. **g**, **h** L929 cells (g) and U937 cells (h) were pretreated with 500 µM MXC for 6 h, 250 µM PXC for 6 h, 4 mM ASA for 24 h or 20 µM DS for 24 h and then treated with 0.5 µg/ml HT‑DNA for 3 h. The cell lysates were immunoblotted with indicated antibodies. **i**, **j** Representative fluorescent images of Hs27 cells stimulated with 0.5 µg/ml HT-DNA for 3 h following a 6 h of pretreatment with 500 µM MXC, indicated by IRF3 (green), p-IRF3 (red) and Hoechst (blue). Scale bar, 10 μm (i). The percentage of cells with nuclear p-IRF3 accounted from 100 views (j). Data are presented as the mean ± s.e.m. ***P < 0.001. Data represent at least three biological independent experiments

Since MXC may play a role downstream from cGAS, we next examined the role of MXC after cGAS activation during DNA stimulation. We performed the immunoblotting to detect the activation of STING, TBK1 and IRF3 after transfection of HT-DNA in both L929 cells and U937 cells. Our data suggested that MXC inhibited the phosphorylation of STING and then led to the suppression of IRF3 phosphorylation (Fig. 4a, b). Consistent results were obtained when treated with cGAMP (Fig. 4c, d). Then, to find out whether MXC’s ability to reduce STING phosphorylation is commonly shared among NSAIDs, we compared the effects with four different NSAIDs, MXC, piroxicam (PXC), aspirin (ASA) and diclofenac sodium (DS). MXC and PXC both belong to the class of enolic acids and are derived from oxicam [19]. Aspirin (ASA), but not diclofenac sodium (DS), was reported to inhibit the IFNβ production through acetylation of cGAS [12]. Our data showed that ASA significantly blocked STING phosphorylation, while PXC and DS did not affect STING phosphorylation (Fig. 4e-f). This data suggested that the inhibitory effect of MXC is unlikely shared among NSAIDs. We next performed the immunofluorescence in Hs27 cells, a human foreskin fibroblast cell line with large cytoplasm, to show the activation of IRF3 by imaging the translocation of phosphorylated IRF3. The results further confirmed that MXC could inhibit the DNA-induced activation of IRF3 in Hs27 cells (Fig. 4g, h). Collectively, our data suggested that MXC inhibits the intracellular DNA-mediated immune responses by inhibiting the STING phosphorylation.

### MXC suppresses the autoimmunity caused by TREX1-deficiency

**Fig. 5:**
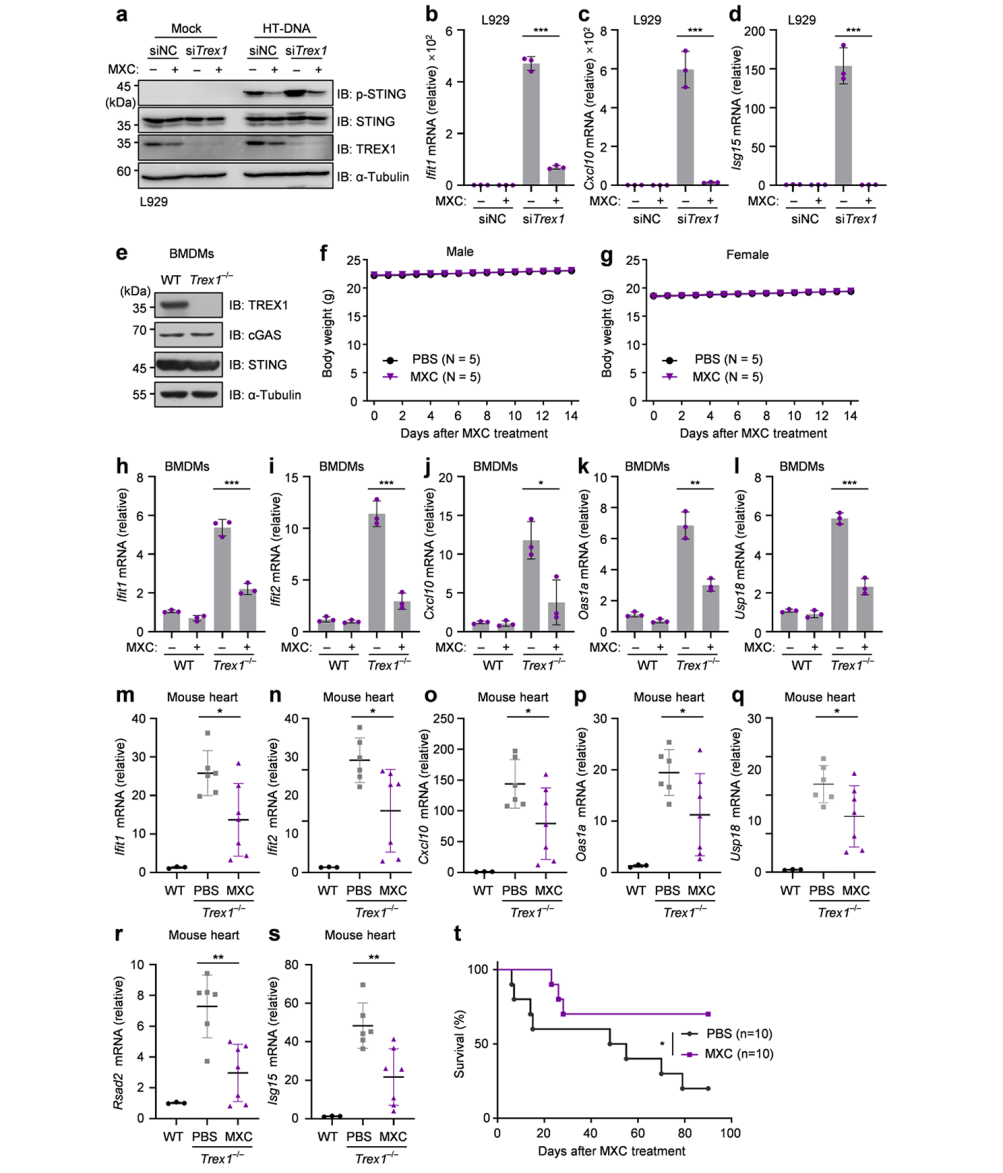
MXC suppresses the autoimmunity caused by TREX1-deficiency. **a** Immunoblotting analysis of indicated proteins in L929 cells after transfected with nontargeting control siRNAs (siNC) or *Trex1*-specific siRNA (si*Trex1*) for 48 h and treated with 0.5 µg/ml HT-DNA for 3 h following a 6 h pretreatment with 500 µM MXC. **b**-**d** qPCR analysis of *Ifit1* (b), *Cxcl10* (c) and *Isg15* (d) mRNA expression in *Trex1*-interfered L929 cells with the treatment of 500 µM MXC for 24 h. **e** Immunoblotting analysis of indicated protein from BMDMs of WT and *Trex1*^–/–^ mice. **f**, **g** Male (f) and female (g) *Trex1*^*–/–*^ mice were treated with MXC (30 mg/kg) daily for 14 days. Body weight was measured every day. **h**-**l** qPCR analysis of mRNA expression of indicated ISGs in WT and T*rex1*^*–/–*^ BMDMs treated with 500 µM MXC for 24 h. **m**-**s** WT mice (n = 3) were treated with PBS and *Trex1*^*–/–*^ mice (n = 6 per group) were given MXC (5 mg/kg, i.p.) for 7 days. Relative mRNA expression of indicated ISGs in mouse heart tissue was measured by qPCR. **t***Trex1*^*–/–*^ mice (n = 10 per group) were given MXC (5 mg/kg, i.p.) or PBS for 90 days. The survival of mice was monitored. Statistical analysis was performed with a two-sided log-rank (Mantel-Cox) test. Data are presented as the mean ± s.e.m. *P < 0.05, **P < 0.01, ***P < 0.001. Data represent at least three biological independent experiments

We next explored whether MXC could be used to treat intracellular DNA-induced autoimmune diseases. We first used a *Trex1* null cell model, widely used to study AGS [10], for further assessing the effect of MXC. A higher phosphorylation of STING was found in TREX1-deficient cells, and MXC effectively inhibited STING phosphorylation in TREX1-deficient cells under the HT-DNA challenge (Fig. 5a). As previously reported, the knockdown of *Trex1* in L929 cells resulted in the increased expression of ISGs [20]. Indeed, the mRNA levels of several ISGs such as *Ifit1*, *Cxcl10* and *Isg15* were upregulated, and MXC inhibited the elevated expression of these ISGs (Fig. 5b-d). In addition, we used the *Trex1*^–/–^ mouse to further confirm the effect of MXC. BMDMs were isolated from both wild-type (WT) and *Trex1*^–/–^ mice and the deficiency of TREX1 expression in mice did not influence the expression of cGAS and STING (Fig. 5e). We then evaluated the potential toxic effect of MXC in *Trex1*^–/–^ mice with daily intraperitoneal injections (i.p.) at a dose of 30 mg/kg for 14 days, and the results did not show any weight loss (Fig. 5f, g), indicating that MXC was well tolerated in both WT and *Trex1*^–/–^ mice. Compared with WT BMDMs, the *Trex1*^*–/–*^ BMDMs increased the expression of several ISGs, while MXC treatment of *Trex1*^*–/–*^ BMDMs decreased the expression of these ISGs (Fig. 5h-l). To further study the effect of MXC in vivo, *Trex1*^*–/–*^ mice and WT littermates received MXC (5 mg/kg, i.p.) daily for 14 days, and the data showed that administration of MXC broadly inhibited the expression of ISGs in heart of *Trex1*^*–/–*^ mice (Fig. 5m-s). Moreover, *Trex1*^*–/–*^ mice exhibited increased survival upon daily administration of MXC (Fig. 5t). Taken together, MXC is effective in treating intracellular DNA-induced autoimmune responses in AGS models.

## Discussion

In this study, we found that MXC attenuated the intracellular DNA-mediated autoimmune response both in vitro and in vivo. We also showed that MXC down-regulated the type I IFN production triggered by different types of DNA. With both human and mouse primary macrophages, we obtained consistent results regarding the effect of MXC. Mechanistic study revealed that MXC affected the phosphorylation of STING downstream of cGAS activation. Importantly, we further demonstrated that MXC was effective in treating intracellular DNA-triggered autoinflammatory responses in *Trex1*^–/–^models and prolonged the survival of *Trex1*^–/–^ mice.

The emergence and accumulation of self-DNA in the cytosol can lead to the persistent activation of cGAS-STING pathway [21]. TREX1-deficiency fails to digest self-DNA in the cytoplasm and thus, the *Trex1*^–/–^ mice exhibit autoimmune and inflammatory phenotypes that are associated with cGAS-STING activation [22]. Previous publications indicated that the deletion of *Cgas* or *Sting* completely rescued the disease phenotypes in *Trex1*^–/–^ mice [23–26], which suggested that the chronic activation of STING is a major player downstream of TREX1-deficiency. Moreover, targeting STING with specific chemical inhibitors could also attenuate the autoinflammatory responses in *Trex1*^–/–^ cells and mouse models [27]. Phosphorylation of STING is an essential mechanism for activating the downstream pathway and the production of type I IFN [3]. Therefore, the development of STING antagonists is efficacious in treating the STING-related diseases [28]. In our study, we reported that the inhibition of STING phosphorylation by MXC could ameliorate the intracellular DNA-mediated autoimmune responses. We noticed that MXC could not affect the phosphorylation of TBK1, which was recruited and activated after the STING trafficking and aggregation [29]. We speculated that MXC may reduce STING phosphorylation through other mechanisms.

NSAIDs are the most commonly used medications worldwide. The primary targets of NSAIDs are cyclooxygenase (COX) enzymes which can produce prostaglandins during inflammatory processes [30]. Although COXs are major targets of NSAIDs to execute the anti-inflammatory function, many studies have reported the COX-independent mechanisms of NSAIDs [12, 31, 32]. Interestingly, we identified a new role of MXC in suppressing the inappropriate production of type I IFN responses by targeting STING phosphorylation. The aberrant activation of cGAS-STING pathway is a major cause for the currently untreatable disease, AGS. Our findings not only expanded the potential indication of MXC, but also provided an immediate translational potential of MXC to AGS.

## Conclusions

By identifying MXC as a potential inhibitor of STING phosphorylation, our study provides a feasible therapy for treating AGS and other autoimmune diseases caused by self-DNA.

## Methods

### Mice

*Trex1*^*+/–*^ mice (C57BL/6) were gifts from D. Barnes and T. Lindahl (Cancer Research UK). All animal experiments were performed in accordance with the National Institutes of Health Guide for the Care and Use of Laboratory Animals and with the approval of the Institutional Animal Care and Use Committee (IACUC-DWZX-2020-511). WT and *Trex1*^*–/–*^ mice (3-week-old) were administrated with MXC (30 mg/kg or 5 mg/kg, i.p.) daily. PBS was used as control. The relative mRNA expression levels of ISGs in mouse hearts were analyzed by quantitative PCR (qPCR). WT mice and *Trex1*^*–/–*^ mice used for in vivo experiments are littermates of heterozygous mating.

### Reagents

Anti-IRF3 (ab68481), and anti-human p-IRF3 (ab76493) were from Abcam; anti-mouse cGAS (31659), anti-mouse p-IRF3 (4947), anti-STING (13647), anti-human p-STING (19781), anti-mouse p-STING (72971), anti-TBK1(3504) and anti-p-TBK1(5483) were from Cell Signaling Technology; anti-TREX1 (611986) was from BD Transduction Laboratories; anti-α-Tubulin (T5168) was from Sigma-Aldrich; anti-IRF3 (sc-9082) were from Santa Cruz Biotechnology. Anti-human GAPDH and anti-human cGAS were prepared and verified in our laboratory. MXC (S1734) and piroxicam (S1713) were from Selleck. Aspirin (50-78-2) was from Ouhe Technology. Diclofenac sodium (D129332) was from Aladdin. PMA (524400), HT-DNA (D6898) were from Sigma-Aldrich; ISD was synthesized from Invitrogen; Poly(I:C) (tlrl-pic), cGAMP (tlrl-cga23) were from InvivoGen; adenosine triphosphate (R0441) and guanosine triphosphate (R0461) were from Thermo Fisher Scientific. The Cy5-DNA (5’-TAAGACACGATGCGATAAAATCTGTTTGTAAAATTTATTAAGGGTACAAATTGCCCTAGC-3’), Cy5-RNA (5’ CGCGACGUGCUCGUACGUGGCUUUGGAGACUCCGUGGAGGAGGUCUUAUCAGAGGCACGU-3’) were synthesized and labeled with a Cy5 fluorophore modifications at the 5′ ends from Tsingke Biological Technology.

### Cell culture and transfection

L929 and U937 were cultured in RPMI-1640 medium containing 10% (vol/vol) fetal bovine serum, 2 mM L-glutamine, 100 U/ml penicillin and 100 mg/ml streptomycin. U937 cells were differentiated with 0.1 µM PMA for 36 h before transfection or other treatment. Hs27 cells and BMDMs were cultured in Dulbecco’s modified Eagle’s medium containing 10% fetal bovine serum, 2 mM L-glutamine, 100 U/ml penicillin and 100 mg/ml streptomycin. L929, U937 and Hs27 cells were obtained from ATCC. All cell lines were regularly tested by PCR for mycoplasma contamination and were found to be negative. BMDMs were differentiated from mouse bone marrow cells with 25 ng/ml recombinant mouse macrophage colony stimulating factor (416-ML, R&D Systems) for 7 days. Transfection of HT-DNA, Poly(I:C), Cy5-DNA, Cy5-RNA and ISD were performed with Lipofectamine 2000 (11668019, Invitrogen) at final concentrations as indicated.

### Cell viability assay

L929 cells were seeded into 96-well plates at a density of 1 × 10^4^ cells per well and incubated with MXC at the indicated concentration for 24 h. The cell viability was analyzed with CellTiter One Solution Cell Proliferation Assay kit (G3580, Promega) according to the manufacturer’s instruction.

### Quantification of cGAMP in cells

L929 cells or PMA-differentiated U937 cells after indicated treatment were performed cGAMP extraction with extraction solvent [40:40:20 (v:v:v) methanol-acetonitrile-water]. Quantification of cGAMP was performed on a triple-quadrupole mass spectrometer (Xevo TQ-S, Waters Corp.) equipped with an electrospray ionization source. The nebulizer gas was 99.95% nitrogen, and the collision gas was 99.99% argon with a pressure of 3 × 10^− 3^ mbar in the T-Wave cell. The gas flows of the cone and desolvation were set as 150 and 800 l/h, respectively. The target compound measurements were performed in the positive mode with a 3.5 kV capillary voltage, 120 °C source temperature and 450 °C desolvation temperature. The optimized ion transitions were: cGAMP m/z 675→524; m/z 675→136.

### Immunoblotting

Cells were lysed with lysis buffer (20 mM Tris-HCl pH 7.5, 0.5% Nonidet P-40, 250 mM NaCl, 3 mM EDTA, 3 mM EGTA, 2 mM dithiothreitol) containing complete protease inhibitor cocktail (04693132001, Roche) and then added by 2 x SDS loading buffer (WB-0081, Dingguo Biotechnology) for 15 min at 105 °C. Cell lysates were separated by SDS-PAGE and proteins were visualized by enhanced chemiluminescence regent according to the manufacturer’s instruction (abs920, Absin).

### qPCR

Total RNAs were isolated from cells using TRI reagent (932879, Sigma-Aldrich). Reverse transcription total RNAs (500 ng) were used by PrimeScript RT Master Mix (RR036A, TAKARA). qPCR was performed with StepOnePlus Real-Time PCR System (Applied Biosystems) by using PowerUp SYBR Green Master Mix (00799448, Applied Biosystems). Data were analyzed with StepOnePlus software. Sequence information of primers are provided in Additional file 1. Human *GAPDH* and mouse *Hprt* were used for normalization.

### PBMCs isolation

PBMCs were isolated from volunteers’ blood after we obtained informed consent. Human primary macrophages were differentiated from PBMCs by using 10 ng/ml recombinant human granulocyte-macrophage colony stimulating factor (215-GM, R&D Systems) for 7 days. PBMCs were isolated from the informed volunteers’ peripheral blood with HISTOPAQUE (1077, Sigma-Aldrich) based on the manufacturer’s instructions and were performed under the approval of the Institutional Ethics Committee (AF/SC-08/02.40).

### Fluorescence imaging

L929 cells were seeded on 96-well plates AND treated with 500 µM MXC for 6 h before being transfected with 0.5 µg/ml HT-DNA for 3 h. Hs27 cells were seeded on coverslips in 24-well plates and treated with 500 µM MXC for 6 h before being transfected with 1 µg/ml Cy5-labeled DNA or RNA for 3 h. The cells were fixed for 15 min with 4% paraformaldehyde, permeabilized with 0.3% Triton X-100 for 10 min, and blocked with 10% BSA for 1 h. L929 cells were stained with Hoechst mounting medium and the images were acquired on the Opera Phenix high-content confocal microscope (PerkinElmer). Hs27 cells on coverslips were then incubated with anti-IRF3 antibody (diluted 1:1000) and anti-p-IRF3 antibody (diluted 1:1000) overnight at 4 °C. Alexa Fluor 488-conjugated and Alexa Fluor 546-conjugated secondary antibodies were applied followed by counterstaining with Hoechst mounting medium. Images were acquired using a ZEISS LSM 880 (ZEISS) confocal microscope.

### RNA interference

For RNA interference-mediated knockdown in L929 cells, the cells were transfected with small interfering RNAs (siRNAs) at a concentration of 40 nM for 48 h. Mouse *Trex1* (MSS238570; 5’-ACCGACAGACUCACAUACUGCUGAA-3’) siRNA was purchased from Invitrogen. Protein expression was analyzed by immunoblotting.

### Statistical analysis

No statistical method was used to estimate the sample size. A standard two-tailed unpaired Student’s t-test was used for statistical analysis of two groups. Statistically analyzed data are presented as mean ± s.e.m. *P* < 0.05 is considered as statistically significant. We performed the statistical analysis by using GraphPad Prism software.

## Electronic supplementary material

Below is the link to the electronic supplementary material.

**Additional file 1.** List of all primers used in the study.

**Additional file 2.** All raw data of immunoblotting assay.

## Data Availability

All data generated or analyzed during this study are included in this article.

## Abbreviations

AGS: Aicardi-Goutières syndrome
BMDMs: Bone marrow-derived macrophages
cGAS: Cyclic GMP-AMP synthase
cGAMP: 2′3′-cyclic GMP-AMP
COX: Cyclooxygenase
Cy5: Cyanine 5
HT-DNA: Herring testis DNA
HSV-1: Herpes simplex virus 1
IFN: Interferon
IFNB: Interferon-beta
i.p.: Daily intraperitoneal
IRF3: Interferon regulatory factor 3
ISD: Interferon-stimulatory DNA
ISGs: Interferon-stimulated genes
MXC: Meloxicam
NSAIDs: Non-steroidal anti-inflammatory drugs
PBMCs: Peripheral blood mononuclear cells
PMA: Phorbol 12-myristate 13-acetate
Poly(I:C): Polyinosinic:polycytidylic acid
qPCR: Quantitative PCR
siRNAs: Small interfering RNAs
STING: Stimulator of interferon genes
TBK1: TANK-binding kinase 1
TREX1: DNA 3’ repair exonuclease 1
VSV: Vesicular stomatitis virus
WT: Wild-type
