# Supplementary figures and images for: Meloxicam inhibits STING phosphorylation and alleviates intracellular DNA-mediated autoimmune responses
Source: Cell Biosci. 2023 Apr 29;13:76. doi: 10.1186/s13578-023-01025-3 (PMC10148517; doi:10.1186/s13578-023-01025-3)

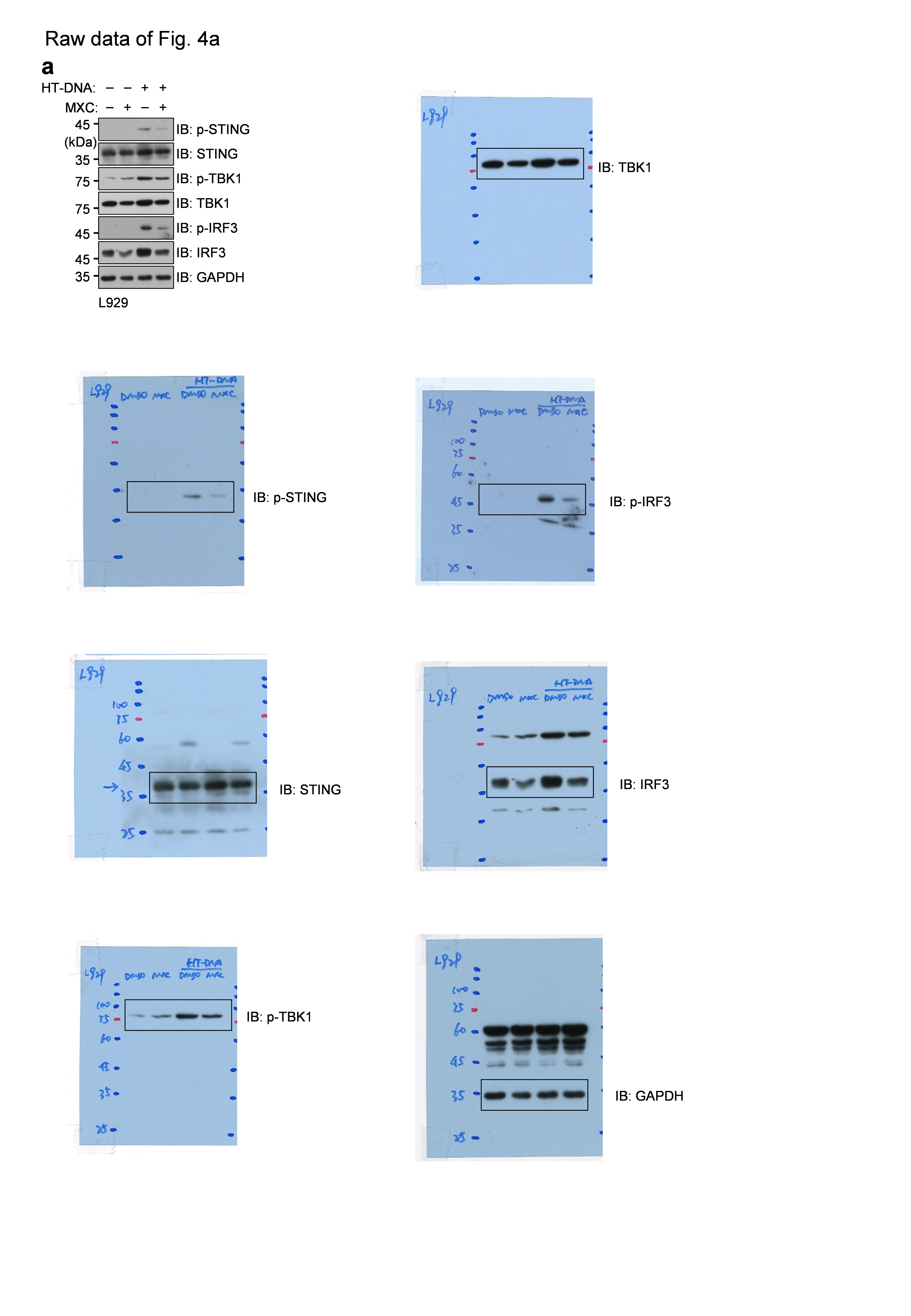


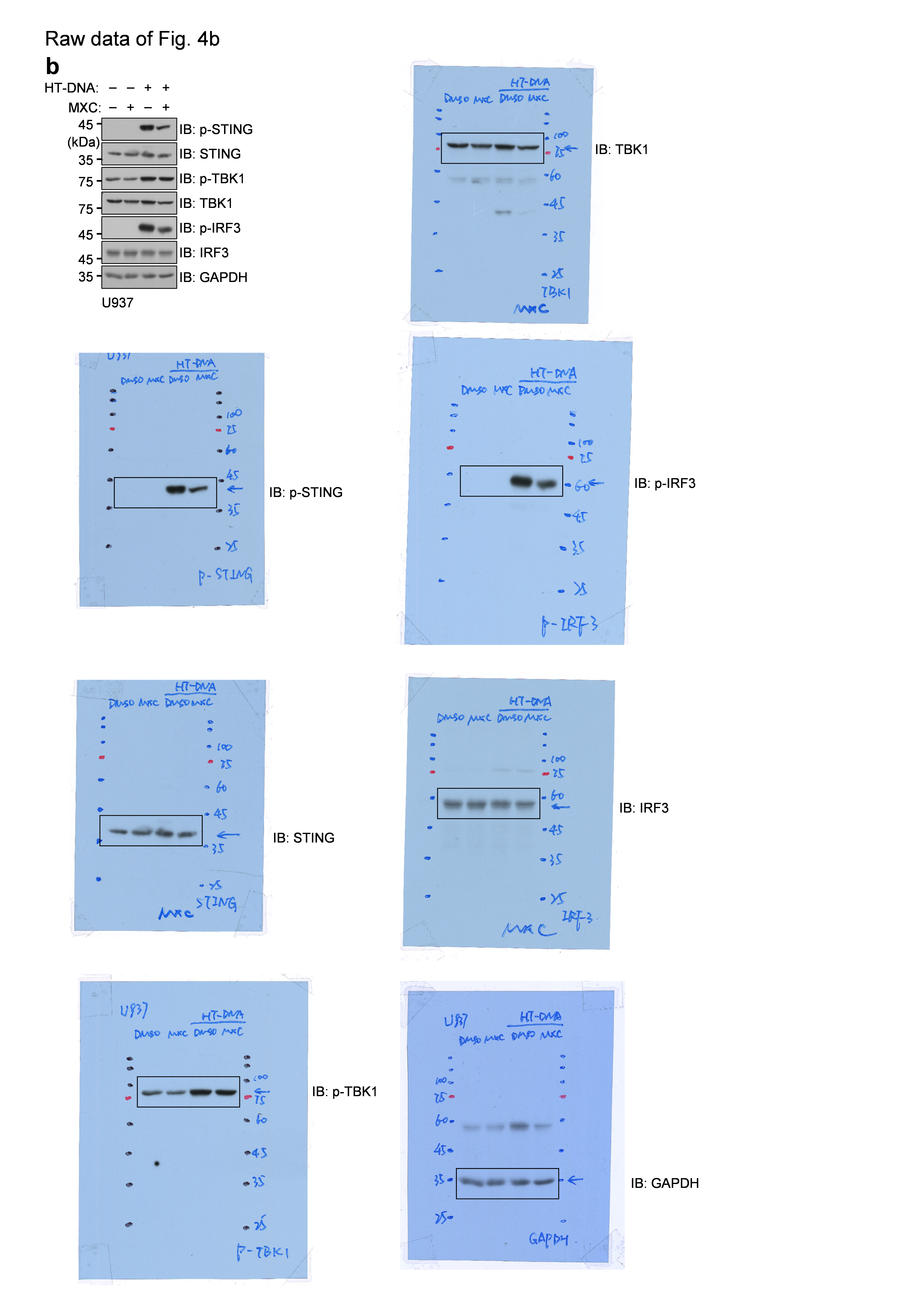


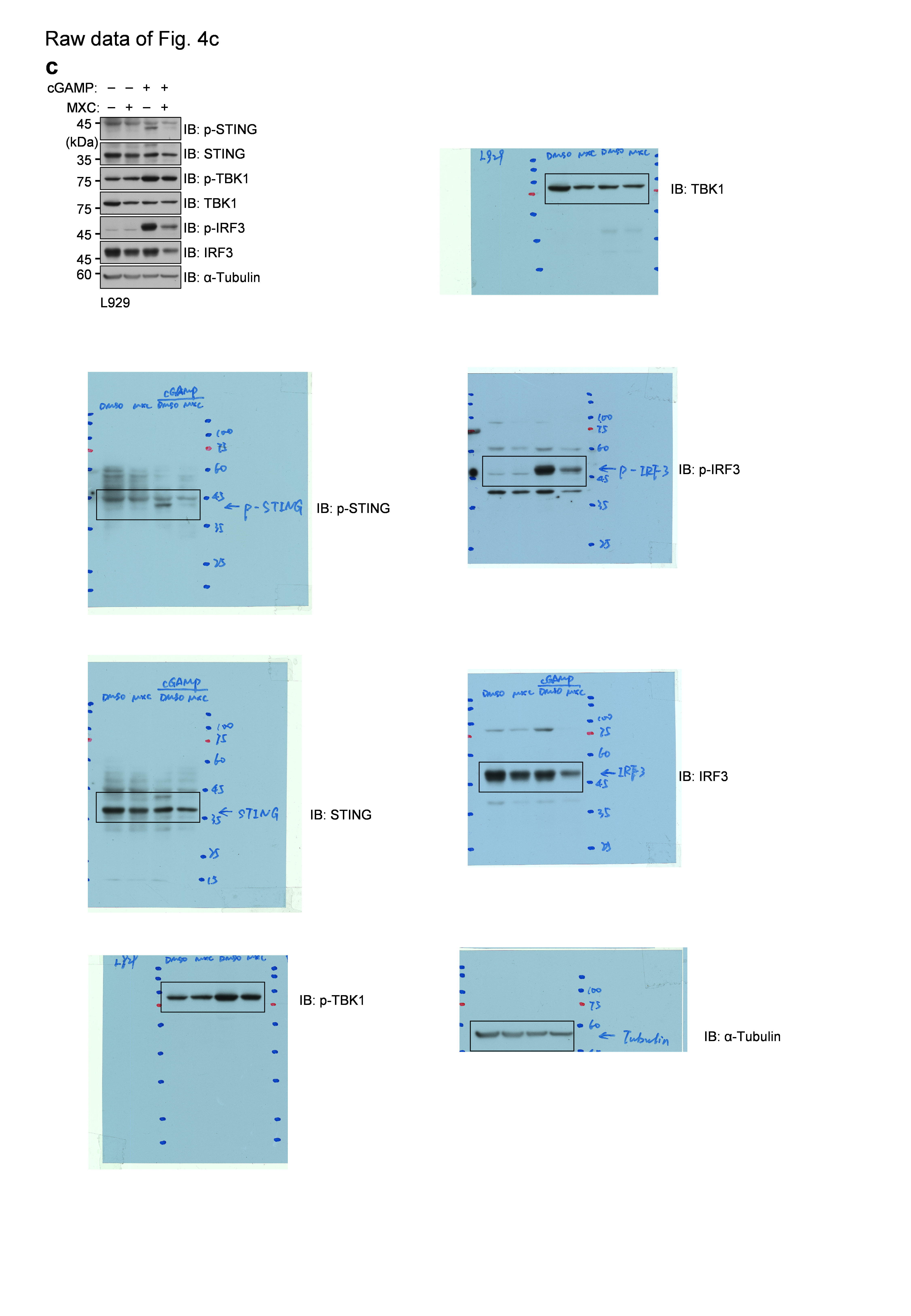


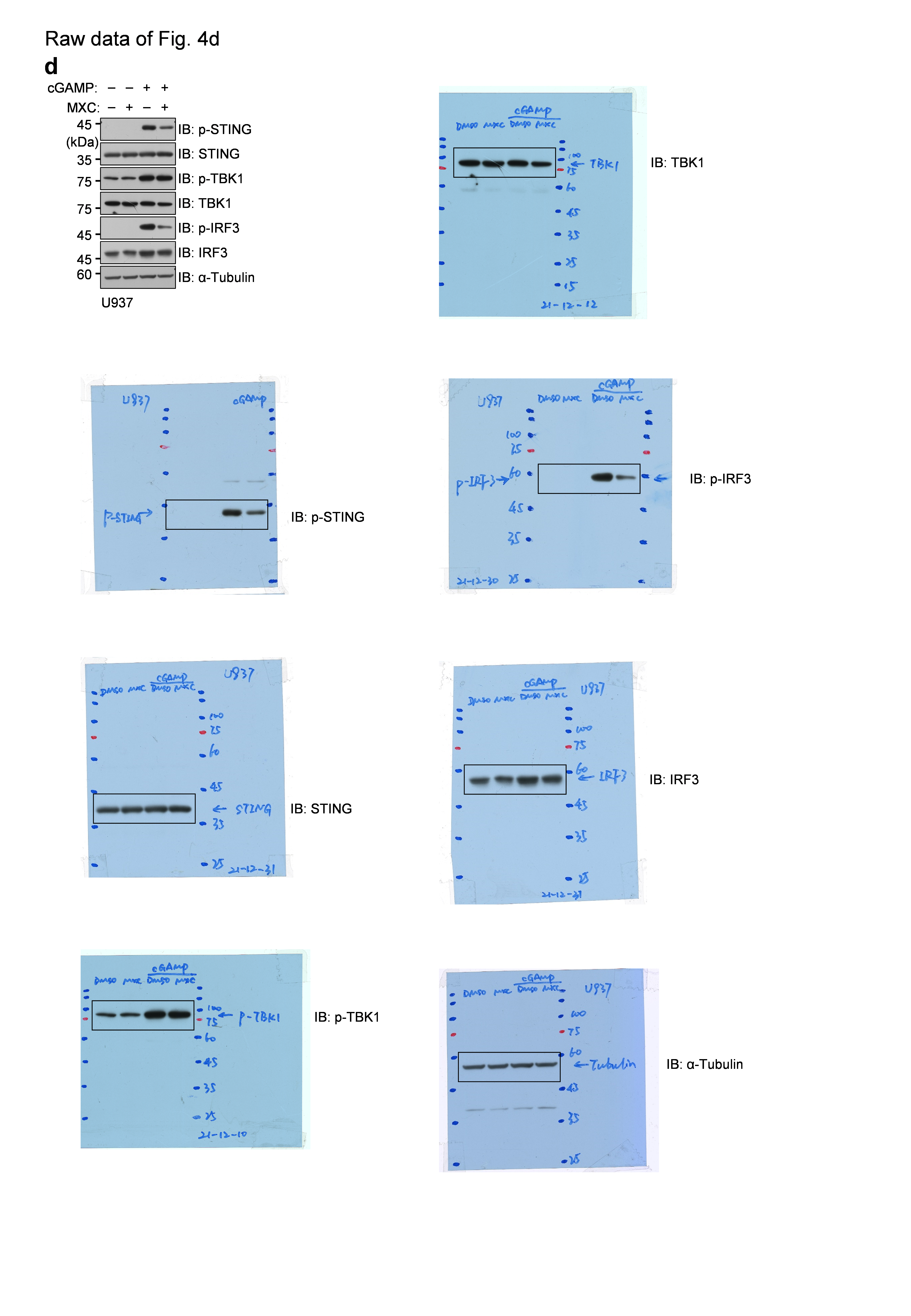


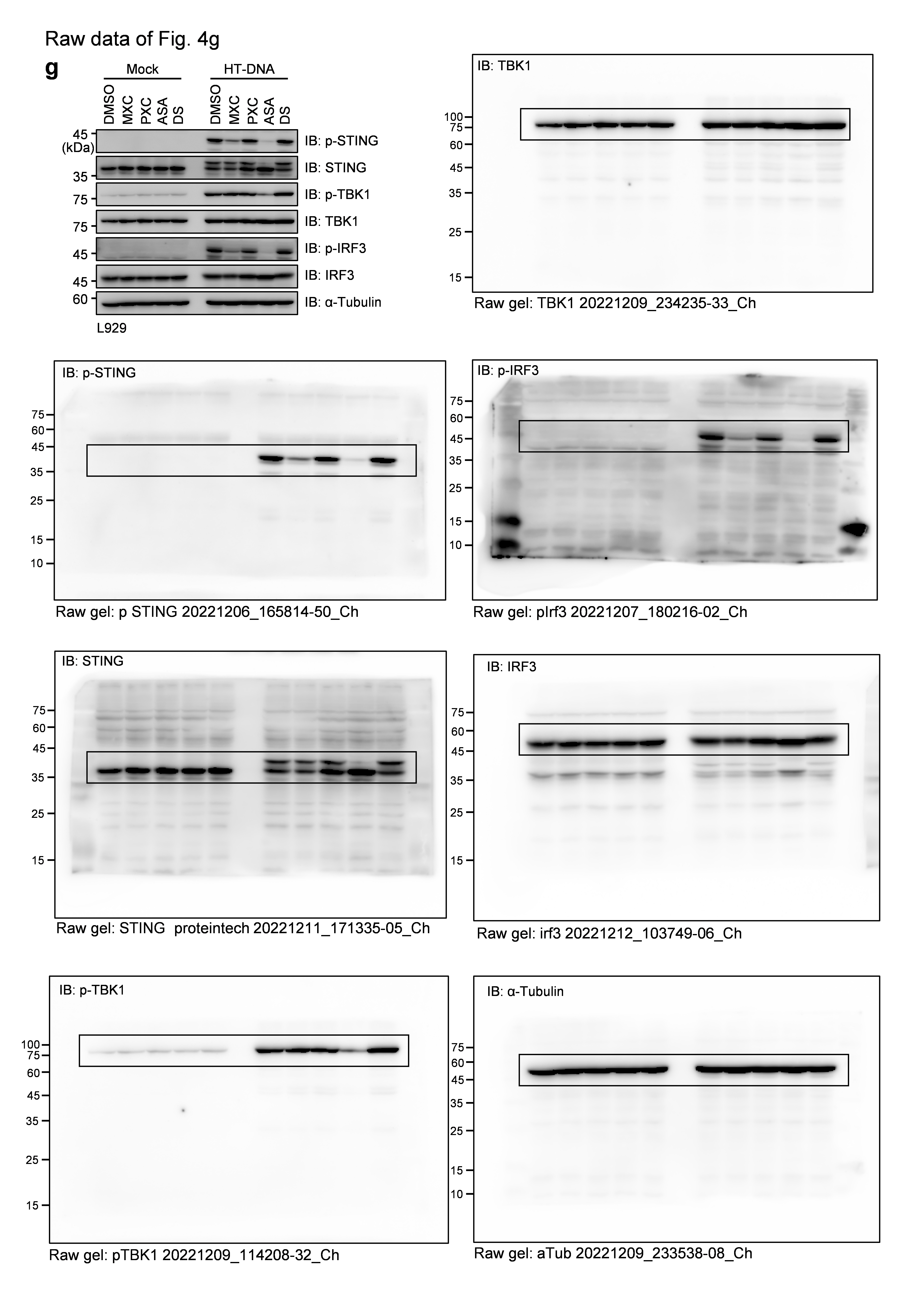


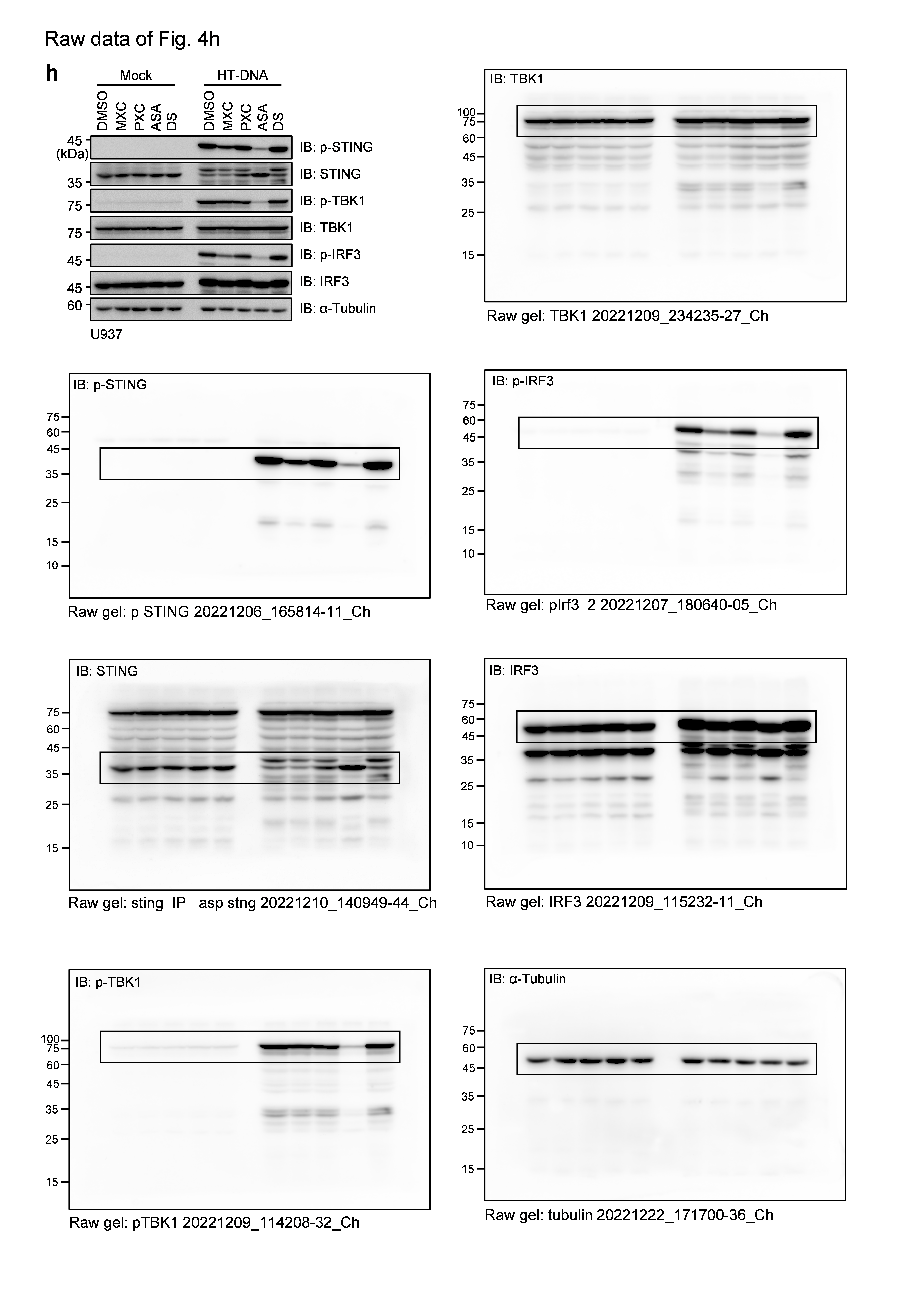


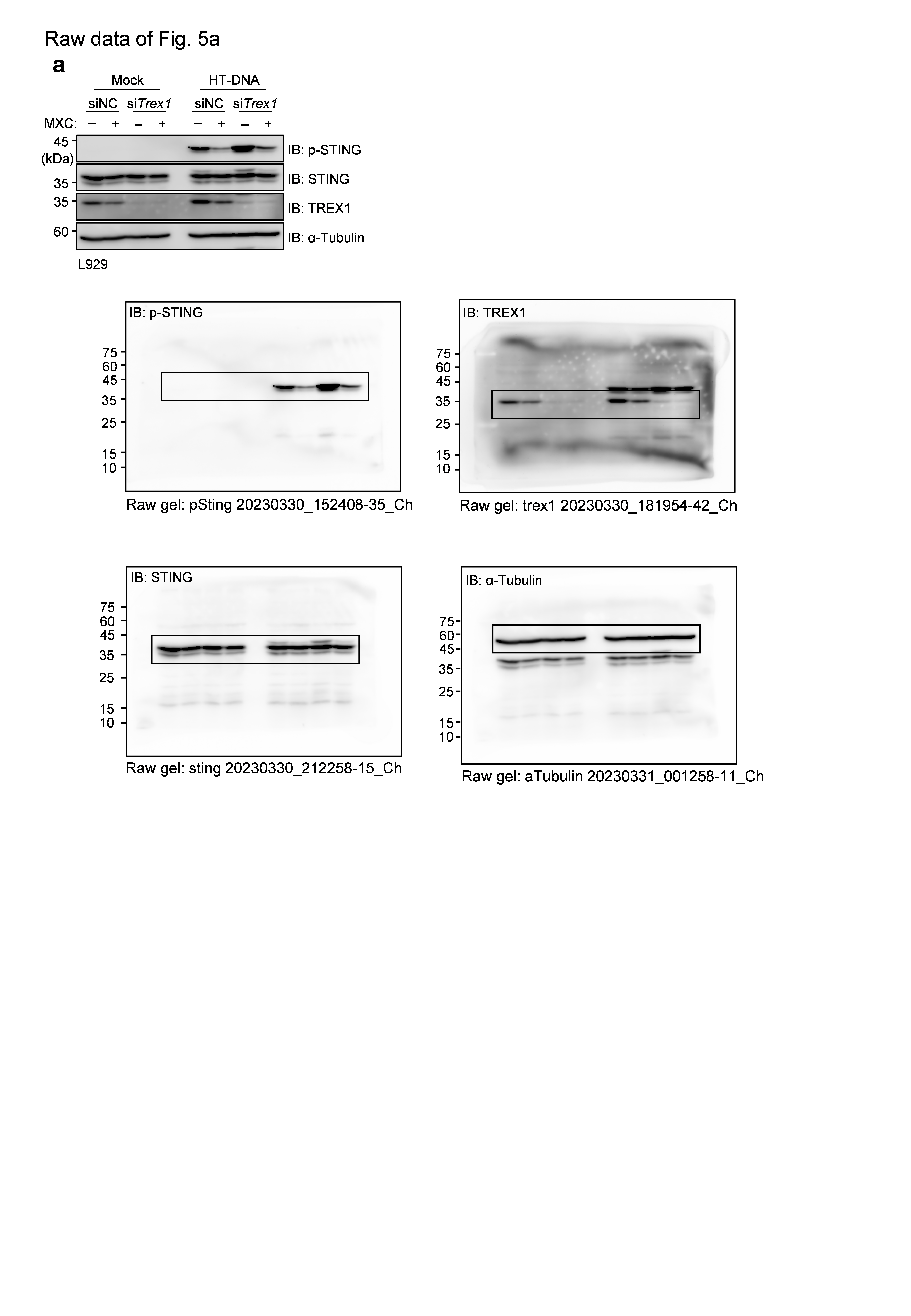


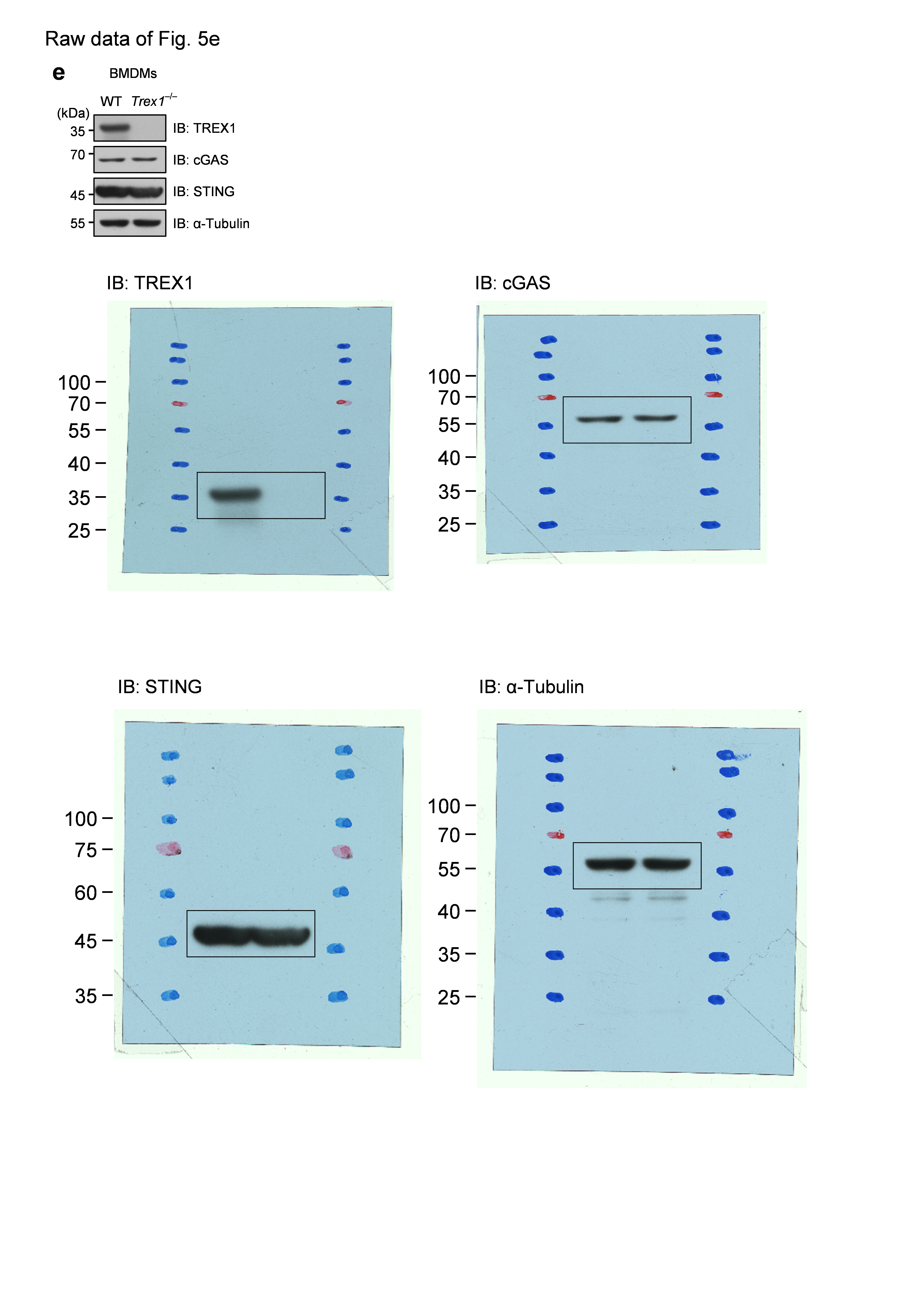

Supplement: Supplementary file 2 — Additional file 2. All raw data of immunoblotting assay. [file 13578_2023_1025_MOESM2_ESM.docx]
